# Salivary Biomarkers in Oral Squamous Cell Carcinoma: A Proteomic Overview

**DOI:** 10.3390/proteomes10040037

**Published:** 2022-11-07

**Authors:** Gabriele Riccardi, Mario Giuseppe Bellizzi, Irene Fatuzzo, Federica Zoccali, Luca Cavalcanti, Antonio Greco, Marco de Vincentiis, Massimo Ralli, Marco Fiore, Carla Petrella, Antonio Minni, Christian Barbato

**Affiliations:** 1 Department of Sense Organs, Sapienza University of Rome, 00161 Roma, Italy; 2 Institute of Biochemistry and Cell Biology (IBBC), National Research Council (CNR), Department of Sense Organs, Sapienza University of Rome, Viale del Policlinico 155, 00161 Roma, Italy

**Keywords:** oral squamous cell carcinoma (OSCC), salivary biomarkers, head neck cancer, proteomics, otolaryngology

## Abstract

Background: Oral squamous cell carcinoma (OSCC) is one of the most frequent cancers worldwide. Endoscopic methods may be useful in the evaluation of oral injuries even though the diagnostic gold standard is a biopsy. Targeted screenings could be considered the best way to prevent the occurrence of oral cancer. Aimed to elucidate the potential identification of specific biomarkers of OSCC, the use of saliva is convenient and noninvasive. Many studies reported more than a hundred putative saliva biomarkers for OSCC, and proteogenomic approaches were fundamental to disclosing this issue. Methods: Relevant literature published in the last few years was systematically searched on PubMed and we focused on articles about the use and study of salivary biomarkers in the diagnostics of head and neck cancer (n = 110). Thereafter, we performed a selection focusing on diagnosis with salivary proteomics in OSCC (n = 8). Results: Saliva proteomics can be a source of biomarkers for OSCC. We reviewed literature of biomarker proteins in saliva that could also be evaluated as probable targets for non-invasive screening of oral neoplasm such as cytokines, matrix metalloproteinases, and acute-phase response proteins. Conclusions: The measurement of salivary biomarkers is a highly hopeful technique for the diagnosis of OSCC. Proteogenomic approaches could permit an accurate and early diagnosis of OSCC. This review seeks to generate an up-to-date view on translational OSCC issues by raising awareness of researchers, physicians, and surgeons. Renewed clinical studies, which will validate the sensitivity and specificity of salivary biomarkers, are necessary to translate these results into possible strategies for early diagnosis of OSCC, thus improving patient outcomes.

## 1. Introduction

Oral cancer is one of the most common cancers worldwide. There are several histologic types with similar incidences, however, the most frequent is oral squamous cell carcinoma (OSCC). The lung, liver, and bone are the main common sites for distant metastases of OSCC, generally detectable only at the advanced stage of the disease. The five-year survival rate of advanced stages of the disease is about 20 to 50 percent, despite improvements in surgical and medical therapies (radiotherapy and chemotherapy) [1]. As yet, even when distant metastases are found at the first stage of OSCC diagnosis, the prognosis is bleak and limited treatment can be proposed.

The most important etiological factors of oral cancer are alcohol, tobacco, and herpes papilloma virus infections [2,3]. Some premalignant lesions are associated with OSCC, and the most common is leukoplakia [4]. In the evaluation of oral injuries, the main diagnostic gold standard is biopsy, in addition to endoscopy. Targeted screenings could be considered the best way to prevent the occurrence of oral cancer and improve public health status, as is performed for breast and cervical cancer [5]. The World Health Organization (WHO) indicates that wide screening is the only effective strategy to lower the morbidity of each cancer, as well as for OSCC. Moreover, with the aim of increasing survival rates of patients with oral cancer, the identification of approved biomarkers as early outcomes is essential in the diagnostic algorithm of OSCC.

In this regard, saliva samples could be considered a useful tool for the study of general clinical conditions due to their numerous molecular contents. Thus, the search for salivary biomarkers could be employed in preventing oral cancer and diagnosing OSCC at an early stage. Saliva is a product of the salivary glands and it contains various proteins, microorganisms, skin cells, serum, and blood derivatives [6]. Cancer-related proteins released by malignant cells and/or other proteins related to various systemic diseases have been discovered in salivary sampling [7,8]. Knowledge of the salivary proteome can help us find potential biomarkers for early oral cancer screening [9].

The present review aims to consider saliva as a diagnostic fluid available for the early diagnosis of OSCC. Furthermore, the detailed literature review of the last decade of proteomic has opened a window on “salivaomics” [10], or the collection of molecules, proteins, miRNAs, epigenomic and genomic changes, and the microbiome used as a diagnostic and predictive tool for many diseases, including oral cancer [11,12,13]. In particular, we will highlight the possible role of salivary proteomics as a crucial screening technique for OSCC [14,15,16].

## 2. Methods

**Figure 1:**
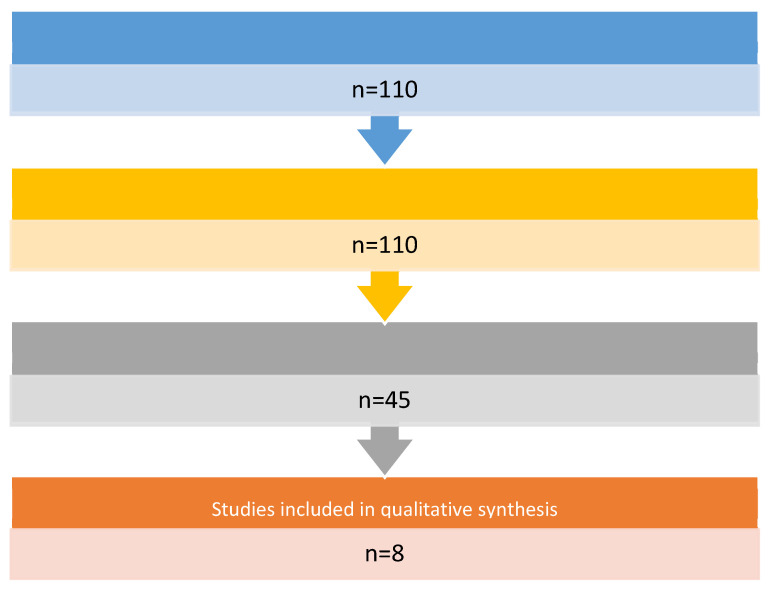
Flow chart of articles selection.

The literature was analyzed through a search in the “PubMed” database using the search terms Proteome * OR Proteomics AND Head and Neck cancer. Limitations on publication date, study design, and language were applied in the search strategy. We have limited the search to reviews, systematic reviews, and meta-analyses, published in the English language over the past decade. The titles and abstracts of the identified records were initially screened and selected by five independent reviewers (G.R., M.G.B., I.F., F.Z., and L.C.) based on their relevance to the review topic. The following set of inclusion criteria, chosen in a shared way, was applied individually to the selected articles in their full-text version: use and study of salivary biomarkers in oncology diagnostics of the head and neck. The literature search produced 110 records (Figure 1). Subsequently, 102 studies were excluded because they did not meet the objective of our review. At least eight studies were included and discussed (Table 1)

## 3. Results

**Figure 2:**
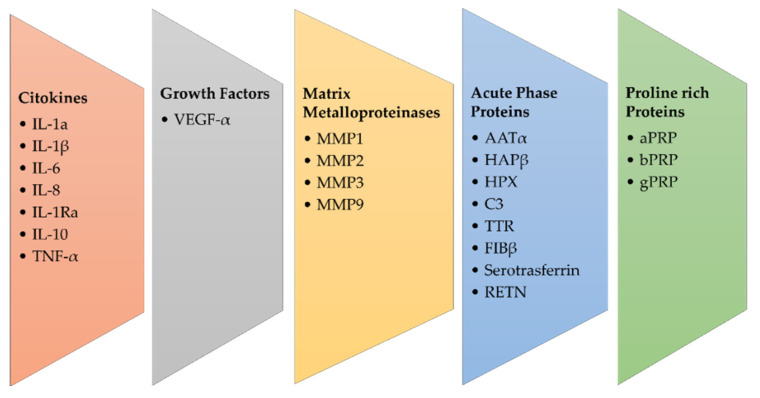
Main groups of salivary biomarkers in OSCC.

Saliva proteomics can serve as a reliable source for biomarker validation for OSCC (Figure 2). Saliva is a promising biofluid that could mirror the physiological and pathological state of the organism [21]. From oral cavity cells and salivary glands, a plethora of proteins are released into the saliva, and their profiling in OSCC is a promising approach. In addition, the liquid biopsy performed on the patient’s saliva is a non-invasive analysis.

Physiologically, the proteins most present in saliva are alfa-amylase, cystatins, pro-line-rich peptides, mucins, and serum albumin. Because more than 50% of cases of OSCC are diagnosed at late stages of disease progression, an early saliva protein content analysis might be a useful strategy to reveal cancer oral disease.

In this regard, the time window of saliva protein analysis could be kept in mind for several studies. In addition, the normal saliva composition with respect to sex, age, and the circadian cycle is a fundamental point. The following reports show a partial view with respect to these mentioned points, but reinforce the opportunity for saliva proteomics.

Cytokeratin has been shown as a potential diagnostic biomarker in head and neck cancer [22]. Salivary levels of Interleukin-1α (IL-1 α), IL-6, IL-8, Vascular-Endothelial Growth Factor- α (VEGF- α) and Tumor Necrosis Factor- α (TNF- α) can support analysis of the progression of premalignant lesions of the tongue and could be used for cancer screening and early diagnosis [23]. Salivary matrix metalloproteinase (MMP) concentrations may be useful for detecting and monitoring OSCC; in fact, as reported by Stott-Miller et al., salivary concentrations of MMP1 and MMP3 were 6–15 times higher in patients with OSCC than in healthy people, with a tendency to increase with higher stage disease [24]. The expression of MMP-9 in saliva has been associated with OSCC and is necessary to obtain an early diagnosis [25].

Shiptzer et al. reported that expression of cyclin D1, Ki67, LDH, and matrix metalloproteinase 9 (MMP-9) were elevated, while measurement of DNA 8-oxoquanine glycosylase (OGG1) and Maspin was lowered in oral cancer saliva patients [26]. MMP2 and MMP-9 are associated with tumor invasion and metastasis, and the concentration of these biomarkers differs significantly in the saliva of healthy individuals, patients with premalignant diseases of the oral cavity, and OSCC patients, suggesting their diagnostic and prognostic role [27,28,29]. In fact, the main protein families involved in salivary proteomics are proline-rich proteins (PRP), including acidic PRP (aPRP), basic PRP (bPRP), and glycosylated PRP (gPRP), α-amylase, mucins, salivary (type S) cystatins, histatins, and statins [30]. Krapfenbauer et al. identified 25 proteins specific to OSCC, suggesting their measurement as biomarkers for oral cancer. Among these, 12 new proteins identified are the proteins galectin-7, cofilin, precursor of CRP, creatine kinase, fatty acid binding protein of the m-chain, type II keratin, myosin light chains 2 and 3, plakoglobin, retinoic acid binding protein II, nucleoside diphosphate kinase A and phosphoglycerate mutase 1 [31].

Starting from the observation that chronic inflammation and oral cancer are connected, acute phase response proteins (APPs) have been detected in the saliva of patients with OSCC, and in particular the APP Haptoglobinβ (HAPβ), α-antitrypsin (AATα), complement-C3 (C3), hemopexin (HPX), serotransferrin, transthyretin (TTR) and fibrinogen β (FIBβ) [31]. In addition, it was suggested that increased levels of HAPβ, AATα, C3, HPX, TF, TTR, FIBβ, and ABG were suitable biomarkers for the early diagnosis of OSCC [31,32]. Yu et al. supported this hypothesis by assembling an inflammatory protein panel for the initial screening of OSCC [33].

Finally, they described another biomarker for the early diagnosis of OSCC, named resistin (RETN), which is a cysteine-rich adipose-derived peptide hormone. Initially, it was considered an endocrine molecule, and after it was associated with type II diabetes mellitus, inflammation, and cardiology disease. RETN has been shown to be highly correlated with the advanced grade of OSCC and metastases. This result highlighted that RETN is a putative salivary biomarker for early diagnosis and is mainly a possible negative prognostic factor of OSCC.

However, the authors are convinced that a standardized system for the collection and analysis of saliva is necessary for it to be used as an early diagnosis target, given the vulnerability of salivary proteins to proteolytic enzymes, oral microorganisms, and the circadian cycle.

### 3.1. Salivary Proteomic Approaches and Methods

An important starting point is the perspective of combining salivary proteomic analysis with standard oral examinations in order to demonstrate the role of salivary proteomic analysis for diagnosis in OSCC (Table 1). In this section, we will describe selected proteomic approaches aimed at biomarker identification in OSCC.

Salivary proteins have been observed to be vulnerable to external factors, and therefore, saliva samples must be collected, refrigerated at 4 °C, and analyzed at a cold temperature to avoid bacterial contamination within 3–6 h. High-speed and high-sensitivity mass spectrometry (MS) allows the investigation of salivary proteomes in a thorough and scrupulous way, also from the point of view of gene expression and post-translational modifications. This approach is usually combined with surface enhanced laser desorption ionization (SELDI), matrix-assisted laser desorption ionization (MALDI) or time-of-flight (TOF) with the aim of measuring intact proteins or peptides (SELDI-TOF-MS) [34].

A biomarker can be defined as a measurable and quantifiable biological parameter that has the ability to play a role in health or environmental exposure and drug responses to a therapeutic protocol. Prognostic biomarkers are used to monitor a benign or malignant condition and give us information about the future development of the health condition, while diagnostic biomarkers indicate the presence or absence of a pathological state, such as cancer or chronic diseases.

Kaczor-Urbanowicz et al. suggest that an integrated electrochemical multiplexing saliva-based platform for oral cancer detection has emerged [17]. This platform is very useful in detecting salivary proteins and nucleic acids such as DNA and RNA, analyzing up to eight different biomarkers in a single session in a few minutes. This saliva test was applied in an Indian cohort of saliva samples in OSCC patients and achieved 90% sensitivity and 90% specificity for both interleukin 8 (IL-8) and IL-8 mRNA. These promising data indicate that this method could be useful for screening and risk assessment for oral cancer and for selecting patients who may need a biopsy [35].

OSCC is common worldwide and, despite improvements in treatment, the 5-year survival rate is disastrous. Factors associated with this failure appear to be late diagnosis and the development of relapses of primary tumors. Therefore, the identification of an OSCC protein biomarker during the initiation and progression of cancer would aid in its diagnosis and treatment by a salivary proteomic approach.

Technologies such as differential gel electrophoresis, two-dimensional polyacrylamide gel electrophoresis, and multidimensional protein identification technology can be used for profiling. Specifically, the use of a small amount of unfractionated serum sample added to a “protein chip”, which is subsequently analyzed by surface-enhanced laser desorption-ionization time-of-flight mass spectrometry (SELDI-TOF-MS) to generate a proteomic signature of the serum, represents an advanced technology [36,37].

Currently, OSCC is diagnosed after a thorough physical examination of the oral cavity looking for signs and symptoms of the disease. When the clinical examination shows an abnormal area in the oral cavity, a tissue biopsy is performed with resection of the same and the verification of the presence of malignant tumor cells by a pathologist. Ishikawa et al. verified that 12 hours after dinner is the best time to collect saliva samples because they report that saliva collected in this time frame expressed significantly different concentrations of metabolites in OSCC patients compared to healthy patients. The study, therefore, suggested a longer fasting period if saliva should be used for diagnosis to improve the discriminatory capacity of any method of analysis [18]. To identify putative protein biomarkers suitable for OSCC detection, all proteins detected in whole saliva samples from patients with OSCC were analyzed. Saliva samples were profiled using rifle proteomics based on C4 reverse phase liquid chromatography for pre-fractionation reverse phase capillary liquid chromatography with quadruple time-of-flight mass spectrometry.

Results demonstrated that the five markers, Mac-2 Binding protein (M2BP), Migration inhibitory factor-related protein 14 (MRP14), CD59 Glycoprotein, catalase, and profilin, provide 90% sensitivity and 83% specificity for OSCC detection [38]. With the aim of identifying biomarkers of cancer development with respect to precancerous lesions, salivary proteins from 12 patients with OSCC and 12 healthy subjects were taken and separated by two-dimensional gel electrophoresis (2DE) and mass spectrometry (MS), and the solid association of α-1-antitrypsin and haptoglobin with OSCC was further enhanced by immunohistochemical staining of tumor tissues [39]. In another similar study, a total of 41 OSCC patients and 30 OSCC-free control subjects were recruited and 2DE and MS methods were used. The sensitivity of the transferrin-based ELISA for oral cancer prediction was 100% for patients with stage T1 oral cancer, 86.6% for stage T2 and 100% for stage T3/T4. This suggests that salivary transferrin may be a biomarker for the detection of the early stage of oral cancer [40]. As for malignant lesions of the oral cavity, a differential proteomic profiling was performed on saliva from dysplastic leukoplakia patients. Initially, 93 proteins were found, and up to 30 overexpressed were selected, and CD44, S100A7, and S100P were significantly associated as putative candidates for the early phase of tumor progression [41]. Aiming to differentiate the early to the late phase of OSCC patients, more recently, Jain et al., individuated from saliva samples several proteins by LC-MS in oral squamocellular cell cancer and thereafter twelve were validated using targeted proteomics [42]. Among these, Alpha 2 Glycoprotein 1 (AZGP1), Alpha-2-Heremans-Schmid Glycoprotein (AHSG), Keratin 6C (KRT6C) and BPI fold-containing family B member 2 (BPIFB2) showed high sensitivity and specificity, suggesting theirs as a potential salivary biomarker of different stages of OSCC [43].

Chattopadhyay et al. summarize the potential of omics studies on salivary biomarkers as diagnostic and prognostic approaches to the detection of oral cancer [12]. The salivary proteome is defined as the complete protein content present in human saliva. Up to two thousand proteins are released into the oral cavity by the acinar cells of all the salivary glands. The salivary proteome has been useful in identifying possible biomarkers for OSCC using advanced proteomics technologies such as mass spectrometry, liquid chromatography, and protein labeling. Endothelin-1 represents a potential biomarker for the development of OSCC in patients with oral lichen planus and IL-8, IL-1β, glycoprotein M2BP (Mac-2 binding protein), CD59, myeloid protein 14 (MRP14) and catalase as salivary biomarkers of oral cancer [44].

Chakraborty et al. summarize all the advances in oral cancer detection and thus also on salivary proteins for OSCC screening and detection [19]. In addition to being non-invasive and inexpensive, the use of saliva has the advantage that sample collection is simple and easy to learn. Ease of storage and transport, sample availability, and ease of repeated collection of salivary samples can improve their feasibility for diagnostic applications [45]. In this article, they are mentioned as molecules for clinical diagnosis in patients with OSCC Cyfra-21 and CA-125, but also mRNA, microRNA, and reactive nitrogen species [46].

Hu et al. analyzed the role of salivary biomarkers in the detection of oral cancer [47]. As well, other authors have shown that saliva contains a collection of analytes, such as proteins, DNA, and mRNA, as well as several metabolites that can be potential biomarkers for clinical and translational applications [48]. Therefore, salivary analysis is an effective option for prevention, monitoring, diagnosis, and prognosis and this biological liquid could even become the first choice for the screening and identification of biomarkers given the constant contact with cancer cells inside the oral cavity [49]. The following section focuses on the cytokines as promising potential salivary clinical biomarkers useful in OSCC.

### 3.2. Salivary Biomarkers in OSCC: The Cytokines

Among proteomic biomarkers in OSCC, the cytokine family is the most important from a diagnostic point of view. Interleukins (IL) play a pivotal role in inflammatory processes and immunity response in malignant transformation, as reported in OSCC [50]. As a consequence, several groups investigated the biomarker role of interleukins in several phases of oral cancer progression, and IL-1-β, IL-6, and IL-8 resulted in potentially predictive salivary biomarkers of OSCC progression [51].

**Figure 3:**
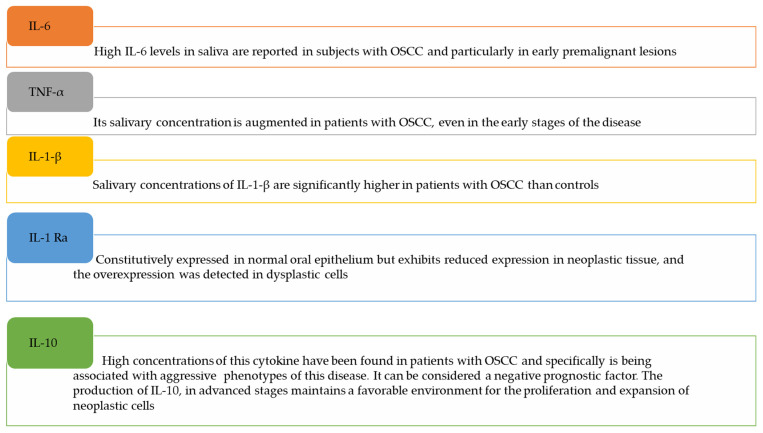
Salivary cytokines as potential biomarkers in OSCC.

Recently, Manzano-Moreno et al. [20] focused their article on the function of cytokines in the diagnosis and prognosis of OSCC saliva, and a revision of this topic was expanded by Ferrari and co-workers [52]. Here we have summarized some significant studies (Figure 3).

*IL-1-β*: salivary concentrations of IL-1-β are significantly higher in patients with OSCC than in controls [53], especially in the early stages of OSCC, suggesting the potential diagnostic value of this cytokine. However, its specificity is lacking because the high salivary concentrations are associated with inflammatory diseases of the oral cavity. On the other hand, Kamatani et al. described a reduction in salivary IL-1β content after surgical resection of the OSCC, strengthening the diagnostic efficacy of IL-1β and its possible use as a diagnostic method [54].

*IL-6*: high IL-6 levels have been reported in the saliva of subjects with OSCC and particularly in early premalignant lesions. IL-6 and TNF-α were associated to distinguish OSCC versus oral leukoplakia. Concentrations of IL-6 and TNF-α have been found to increase exponentially with the progression of OSCC, given their pathophysiological role in the survival and proliferation of malignant cells [55,56,57].

*IL-8*: It is extensively described in the literature that there are elevated salivary concentrations of IL-8 in patients with OSCC. As for the aforementioned cytokines, it is supported by the fact that they control the growth and proliferation of tumor cells, favoring their escape from immune defense actions. In patients with premalignant lesions such as lichen planus, oral leukoplakia, and oral submucosal fibrosis, elevated concentrations of IL-6 and IL-8 can be found in the saliva and serum, and furthermore, their high levels in biological fluids are correlated with reduced survival and increased relapse rate in OSCC, if previously diagnosed [58].

*IL-1-Ra*: Niklander et al. found that, in contrast, IL-1-Ra is constitutively expressed in the normal oral epithelium but exhibits reduced expression in neoplastic tissue. The overexpression of IL-1-Ra, on the other hand, was detected in dysplastic cells together with the overexpression of IL-1 β [59]. Shiiba et al. reported that IL-1-Ra had a sensitivity of 70% and a specificity of 85% to discriminate between OSCC and other potentially malignant oral diseases such as lichen planus [60].

*IL-10*: high concentrations of this cytokine have been found in patients with OSCC and specifically have a negative prognostic factor, being associated with aggressive phenotypes of this disease. From the pathophysiological point of view, the production of IL-10 in advanced stages of metastatic tissues maintains a favorable environment for the proliferation and expansion of neoplastic cells [61,62].

*TNF-alpha*: its salivary concentration is augmented in patients with OSCC, even in the early stages of the disease. In this way, it is useful for differentiating between OSCC and oral leukoplakia or other pre-malignant lesions. Krishnan et al. showed that TNF-α in the saliva of patients with stage IV OSCC compared to patients with stages I, II, or III, was overexpressed, supporting its association with advanced stages of pathology [63].

Brailo et al. found that IL-6 and TNF-α levels in saliva were higher in patients with oral leukoplakia [57]. Furthermore, Rhodus et al. reported that the levels of IL-1, IL-6, IL-8 and TNF-α were significantly higher in the saliva of patients with oral cancer than those of patients with oral dysplasia [64].

Therefore, saliva can be used to screen the entire population for disease progression, and the application of salivary biomarkers will be essential for early diagnosis and good prognosis, with the aim of reducing mortality associated with OSCC.

In this way, the analysis of salivary proteomics will play a central role not only in the diagnosis but also in the clinical treatment of oral cancer [65], and the proteomic ILs dosage could be an effective strategy.

## 4. Discussion

We aimed to point off an overview of selected studies focused on proteomic analysis of potential saliva biomarkers in OSCC. “Omic” approaches are largely used in the diagnosis of head and neck cancer and oral squamous cell carcinoma, and saliva represents a source of non-invasive biological samples [66]. Current experimental approaches aimed at screening, early diagnosis, and individuation of prognostic factors for oral malignant lesions are few and ineffective [67].

The evolution of biomarker-targeted salivary diagnostics and “salivaomic” diagnostics lays the foundation for the development in the future, even of small portable devices capable of early detection of oral cancer in a non-invasive, cost-effective, and rapid manner [68]. Several emerging saliva-based technologies have already demonstrated their potential clinical utility, and if properly implemented in the near future, the diagnostic tools will be used successfully by physicians for the early detection of oral cancer.

The aim is obviously the timely diagnosis of premalignant lesions because early-stage OSCC is associated with higher survival rates and therefore, ultimately with an improved prognosis. It is obvious that a single biomarker may not be sufficient and sensitive enough for the differentiation between oral carcinoma and normality. More sensitive and specific biomarkers may be needed to screen high-risk patients in addition to serum biomarkers.

Specifically, the measurement of oral cytokines in the saliva is among the most promising techniques for diagnosing and evaluating the prognosis of OSCC. In particular, the combined saliva expression of IL-1-β, IL-6, and IL-8, among many other biomarkers evidenced from the literature, represents a valid “trio” in terms of sensitivity and specificity of OSCC diagnosis. Recently, a meta-analysis on IL-6 and IL-8 biomarkers in OSCC suggested that they are predictive of OSCC [69]. To overcome the problem of increasing the wide involvement of cytokines in inflammatory conditions, it could be important to have a combination of proteomic data, including oral cytokines and salivary miRNAs, for an accurate and early diagnosis. In addition to these biomarkers, small molecules, such as non-coding RNA [70] and metabolite analysis [48], could represent a second group to build the construction of a salivary biomarker panel for OSCC.

Furthermore, it should be considered that these salivary biomarkers, together with the analysis of other histopathological markers (eosinophils) and the immune profile, could be the basis for the development of new strategies in targeted therapies.

## 5. Conclusions

Although some of these biomarkers have been detected, it is important to implement them by finding other proteins among the many that have shown high potential, all of which indicate and develop clinical applications. New clinical studies, which will validate the sensitivity and specificity of these biomarkers in clinical settings, are needed to translate these findings into potential strategies for early detection, leading to improved patient outcomes. The validation of selected biomarkers and biomarker panels in large patient cohorts is required before they can be used in clinical practice.

Human saliva is a unique biofluid with huge clinical and diagnostic potential. It represents a unique opportunity for medical studies on head and neck pathology, combining non-invasive and low-cost analysis. The “salivaomic” profile, as a result of transcriptome, proteome, metabolome, microbiome, and exosome integration data analysis, is a very promising field of investigation to search for translational biomarkers.

We expect significant signs of progress in the near future, and suggest that proteogenomic approaches, as the interplay between genome and proteome, may be a promising life science translational research field.

## Figures and Tables

**Table 1:** Selected studies on Proteomic and OSCC.

Author	Country	Institutional Affiliation	Method	Purpose
Aro et al. [10]	Finland	University of Helsinki	Genomics; Proteomics and transcriptomics; Lipidomics; Metabolomics; Microbiomics	Review of the recent advancements in the field of salivary diagnostics in oral cancer
Li et al. [11]	China	Sichuan University	Salivary proteomics for oral cancer	Putative salivary proteomic biomarkers in oral cancer screening
Chattopadhyay et al. [12]	India	Central University of Tamil Nadu	GSTT1 Polymorphism in Salivary DNA; Role of circulating tumor DNA (ctDNA); Salivary methylome; Salivary transcriptome; Salivary Proteomics, Salivary Microbiome; Salivary Metabolomics	Saliva biomarkers as diagnostic and prognostic tools
Ni et al. [15]	China	Nanjing University	Protein biomarker	Early detection by tissues, salivary and serum
Kaczor-Urbanowicz et al. [17]	USA	University of California at Los Angeles	Poc diagnostics; RNA sequencing; Liquid biopsy; Electromagnetic field-based techniques; Electric field stimulates release and measurement method	Introduce population-based screening programs
Khurshid et al. [18]	Saudi Arabia	King Faisal University	Whole-Mouth Saliva Biomarker	Non-invasive diagnosis
Chakraborty et al. [19]	India	Vellore Institute of Technology	Microfluidics systems Salivary biomarkers (Telomerase, HPV16 DNA, Reactive nitrogen species and antioxidants, Salivary vitamin E and C)	Early stage of diagnosis
Manzano-Moreno et al. [20]	Spain	University of Granada	Saliva, MicroRNA and Cytokines	Diagnosis and prognosis of OSCC

## Data Availability

Not applicable.
